# Overview of Aging, Skin Health, Estrogen, Menopause and HRT

**DOI:** 10.3390/life16030401

**Published:** 2026-03-02

**Authors:** Edwin D. Lephart, Zoe D. Draelos

**Affiliations:** 1 Department of Cell Biology, Physiology and The Neuroscience Center, College of Life Sciences, Brigham Young University, Provo, UT 84602, USA; 2 Dermatology Consulting Services, PLLC, High Point, NC 27262, USA

**Keywords:** aging, skin, estrogen, perimenopause, menopause, HTR, MHT

## Abstract

While skin aging is inevitable, healthy habits, sun protection, skincare, and medical interventions can slow visible skin changes; estrogen is also crucial. In 2002, the Women’s Health Initiative (WHI) results contributed to the subsequent trend toward fear and avoidance of hormone replacement therapy (HRT). Since 2002, the WHI results have been re-evaluated and caused the US FDA to announce “the removal of the misleading FDA warnings on HRT”, stating that “estrogen is a key hormone for women’s health where every single part of a woman’s body depends on estrogen to operate at its best—including the brain, bones, heart, and muscles”. This overview explores this transformation of scientific information/perspective on HRT via (a) aging and skin health; (b) the importance and changes in estrogen in women with a focus on dermal parameters; (c) provides a brief review of the WHI result, impact, and current status of this report; (d) explores the “timing hypothesis” for HRT interventions; and (e) proposes that HRT might be considered not only for symptomatic women but also for esthetic treatment in perimenopause and menopause patients. The latest reviews support a recent large-scale systematic review and meta-analysis on skin parameters, which suggests that HRT may have a place in esthetic treatment. However, beyond esthetic benefits, the positive implications of HRT on several other health parameters in women during aging are briefly presented. Of course, hormonal and numerous other treatments require a review of the risks/benefits and their discussion among the patient and medical professionals to determine the most effective interventions for treating hormone-related skin changes, but this shift in perspective warrants further investigation and validation.

## 1. Introduction

The purpose of this narrative overview is to present the topics of aging, skin health, estrogen, and menopause in connection with an examination of the transformation and progression of scientific evidence regarding the Women’s Health Initiative (WHI). The WHI was a landmark study that revolutionized women’s health, resulting in a massive, immediate decline in hormone therapy use and a shift in global clinical practice away from hormone replacement therapy [HRT] [1]. This overview focuses on today’s perspective, especially concerning the re-evaluation of the vital roles of aging and estrogen as essential functions in women’s health, to provide awareness and address the progressive decline in women’s wellbeing during menopause, where estrogen plays a key role in enhancing brain, bone, heart, and muscle health with aging [1,2]. Specifically, estrogen provides critical, comprehensive protection to women by promoting cardiovascular health (lowering LDL, raising HDL, and aiding vasodilation), maintaining bone density, enhancing cognitive function and neuroprotection (particularly against neuroinflammation, which can lead to neurodegenerative diseases), and enhancing skin parameters to prevent infections and regulate metabolism [2]. Thus, this overview explores the transformation of scientific information/perspectives on HRT by covering (a) aging and skin health; (b) the importance of and changes in estrogen in women, with a focus on dermal parameters; (c) a brief review of the WHI results, impact, and current status on this report; (d) the “timing hypothesis” for HRT interventions; and (e) a proposal that HRT might be considered not only for symptomatic women but for esthetic treatment in perimenopause and menopause patients.

This narrative overview uses foundational figures, graphics, and tables that depict results from the recent literature covering the topics listed above, mainly from the last six years (from January 2020 to January 2026), with general and more recent reports included where applicable. These topics were explored using the following keywords: aging, skin aging, skin health, estrogen, menopause, HRT, WHI, and current perspectives on WHI. The following databases were used: PubMed, Science Direct, Scopus, and Google Scholar. Some references were included without a year interval range limit, which provided data and background information on various topics, such as the historical, biochemical, and molecular mechanisms for the factors searched. We addressed the limitations of selection and interpretation bias by defining the factors, concepts, and principles investigated and by capturing the most up-to-date research available to present a clear understanding of the themes and hypotheses covered in this narrative overview. In general, references focused mainly on human investigations that included observational studies, comprehensive reviews, systematic reviews, and meta-analyses where accessible and appropriate.

## 2. Aging and Skin Health in Women

**Figure 1:**
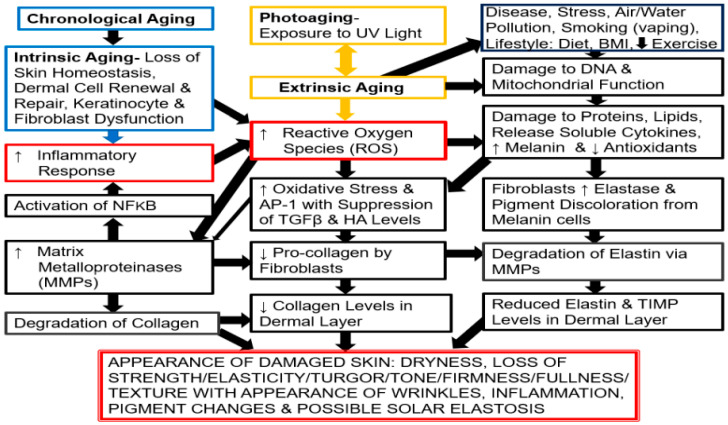
Chronological aging via the loss of skin homeostasis/oxidative metabolism, photo-aging by exposure to UV light, and extrinsic aging (due to external factors and lifestyle) through cellular/molecular signaling mechanisms are shown. The cascade events, including the major impact of oxidative stress by the generation of reactive oxygen species (ROS) is displayed in reference to the appearance of damaged/inflamed skin and wrinkles due to changes in dermal structural proteins (collagen and elastin). Pro-inflammatory transcript factor NFkB (NFkb), AP-1, a nuclear transcription element, Activator Protein-1 (AP-1), hyaluronic acid (HA), Tissue Inhibitor of Matrix Metallo-proteinase (TIMP), and Transforming Growth Factor beta (TGFβ). Adapted from  Ref. [18], license CC BY 4.0. Up arrow indicates increase; down arrow indicates decrease.

Aging is a gradual biological process influenced by genetics, hormones, lifestyle, and environmental factors [3,4,5,6]. The skin is the most conspicuous organ to display signs of aging or changes in dermal health due to chronological (intrinsic) and photo-aging (extrinsic) mechanisms [7,8,9], which are shown in Figure 1. Unlike other organs, the skin is exposed to the external environment, especially in areas like the face, neck, hands, and arms [7,8,9].

While comprehensive coverage is not the purpose of this overview, skin aging is presented in brief, which is an important component in cosmesis, appearance, diagnosis, and management of the skin [7,8,9,10,11]. Intrinsic or chronological aging goes beyond the passage of time and incorporates several factors such as genetics, metabolic, hormonal, immunological, cardiovascular, gastrointestinal, degenerative, and neoplastic diseases, along with psychogenic conditions (involving stress or affective disorders) [7,8,9,10,11,12,13,14,15,16,17,18].

However, one of the most important factors in chronological (intrinsic) and photo-aging (extrinsic) aging is the generation of oxidative stress, which is the imbalance between reactive oxygen species (ROS) and the skin’s ability to neutralize them with antioxidants like catalase, superoxide dismutase, and glutathione. Notably, ROS are a normal byproduct of cellular metabolism within mitochondria, and as we age, they accumulate faster than they can be neutralized. As shown in Figure 1, ROS associated with chronological (intrinsic) aging lead to skin damage affecting various dermal components [7,8,9,10,11,13,14,15,18]. Conversely, photo-(extrinsic) aging is due to exposure to the sun (ultraviolet light), which is the major factor in skin aging because it involves acute or sustained exposure that results in bursts of ROS. Photo- or extrinsic aging is more severe compared to chronological or intrinsic aging because it activates a cascade of inflammatory events with harmful molecules and causes skin damage, as outlined in Figure 1 [14,18].

If skin changes in women are examined starting at age 20 until age 60 and beyond, there is a progressive decline in dermal health [7,8,9,10,13,14,18]. This is due to oxidative stress not only with chronological (intrinsic) aging but also with photo-(extrinsic) aging [7,8,9,14,15,16,18]. Thus, structural changes in skin begin in the mid-20s and accelerate during perimenopause and menopause due to the decline and loss of estrogen from the ovaries, which plays a major role in collagen and elastin production [7,9,18] (see Section 3 below). Additionally, there is decreased wound healing with thinner, drier, and less elastic skin [7,8,9,10,11]. Moreover, subcutaneous fat loss and redistribution alter facial contours, leading to sagging and more fragile epidermal thinness that increases the risk of injury [8,9,10,11,13]. Furthermore, skin moisture and barrier function decline, leading to irritation and infection [8,9,13,14,15,16,17]. Finally, uneven melanin distribution causes age spots (hyperpigmentation), and dull and uneven skin tones occur with aging [7,8,9,14,17].

## 3. The Importance of Estrogen and Changes in Estrogen During Aging

**Figure 2:**
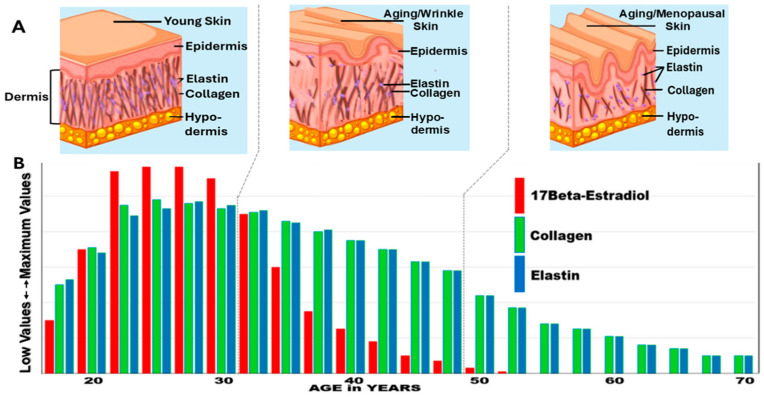
(**A**) The upper panel displays the progressive decrease in dermal collagen and elastin fibers and the appearance of fine lines and wrinkles with the passage of time. (**B**) The lower panel displays the ovarian 17β-estradiol levels (red histogram bars) in women from the late teenage years to seventy years of age. After menopause, low estrogen levels (not shown) are derived from the aromatization of androgens in peripheral fat tissue sites. The profile of collagen expression in skin with aging is displayed (green histogram bars), while the elastin levels (blue histogram bars) follow the pattern of ovarian estrogen production with aging. Adapted from Ref. [18], license CC BY 4.0.

Estrogen plays a critical role in women’s health, particularly with aging, as its gradual decline during perimenopause and subsequent loss at menopause significantly affects multiple aspects of skin health [7,8,9,13,14,15,16]. This is attributable to the widespread presence of estrogen receptors across nearly all cells in the body, influencing most tissues and organs and contributing to numerous age-related disorders [7,9,19,20]. Estrogen is well recognized for its role in maintaining key skin components, including the extracellular matrix, collagen, and elastin [7,8,9,18,19,20]. As estrogen levels decrease, there is a corresponding reduction in collagen and elastin, beginning in early adulthood, at an estimated rate of approximately one percent per year [7,8,9,18,19], as shown in Figure 2. This decline continues through perimenopause, which typically occurs between 40 and 50 years of age [7,8,9,19]. At menopause, generally around age 50, ovarian estrogen (i.e., 17β-estradiol) production ceases, resulting in an approximate 30 percent loss of collagen within the first five years, followed by an additional annual decline of about two percent thereafter [7,8,9,19], as shown in Figure 2.

In addition to the changes in estrogen, collagen, and elastin levels, there is also a range of other dermal structural and functional parameters influenced by estrogen, including the microbiome [21,22,23] and facial attractiveness/perceived self-perception [24,25], which are listed in Table 1.

## 4. The Women’s Health Initiative (WHI) and Perspective on Hormonal Replacement Therapy

Prior to the Women’s Health Initiative (WHI) report in 2002, hormone replacement therapy (HRT) was widely prescribed and strongly endorsed not only for the management of menopausal symptoms, but also as a preventive intervention to reduce the risk of cardiovascular disease, osteoporosis, and cognitive decline, and to support overall health [1,26,27,28,29,30]. Prescribing practices frequently included women without significant menopausal symptoms, and many women remained on HRT for prolonged periods, often spanning years or decades [1,26,27,28,29,30]. Both estrogen-only therapy (for women without a uterus) and combined estrogen–progestin regimens were commonly used [1,28,29,30].

The initial WHI findings, reported in 2002, which were not stratified by age, demonstrated that combined oral conjugated equine estrogen (CEE) and medroxyprogesterone acetate therapy initiated an average of ten years after menopause was associated with increased risks of breast cancer, stroke, venous thromboembolism, and coronary heart disease [1,28,29,30]. Estrogen-only therapy was associated with increased risks of stroke and thromboembolism without evidence of cardiovascular benefit [1,28,29,30]. Following the publication of the WHI results, HRT use declined dramatically worldwide. Many women discontinued therapy abruptly, and media coverage largely emphasized risks without adequately accounting for the age of participants or the extent of pre-existing disease in the WHI population [1,28,29,30]. Consequently, HRT was no longer recommended for chronic disease prevention and was reframed as a symptom-directed therapy primarily for vasomotor symptoms (e.g., hot flashes and night sweats) and genitourinary syndrome of menopause [1,28,29,30]. Clinical guidance emphasized the use of the lowest effective dose for the shortest duration necessary, generally in symptomatic women younger than 60 years or within 10 years of menopause onset [1,29,30].

Subsequent analyses demonstrated that the risks and benefits of HRT are age- and time-dependent, with more favorable risk profiles observed among women who initiate therapy closer to the onset of menopause [1,29,30]. Additionally, transdermal estrogen and lower-dose formulations appear to mitigate certain risks. In summary, whereas HRT was broadly prescribed as a long-term health-promoting therapy prior to the WHI report, post-WHI report, use has become more cautious, selective, and primarily focused on symptom management rather than disease prevention [1,26,27,28,29,30].

Since 2002, the WHI results have been re-evaluated (by a comprehensive review of scientific evidence, expert panel input, and public commentary) and caused the US FDA to announce on 10 November 2025 “the removal of the misleading FDA warnings on HRT”, stating that “estrogen is a key hormone for women’s health where every single part of a woman’s body depends on estrogen to operate at its best—including the brain, bones, heart, and muscles” [31]. Table 2, below, summarizes the characteristics of the WHI report from 2002 compared to today’s perspective (2020s) [1,2,28,29,30,31,32,33].

Therefore, the US FDA’s action reflects a broader shift in medicine toward evidence-based, nuanced regulation that (a) recognizes scientific advances since the early WHI studies, (b) reduces barriers created by overly cautious historical labeling, and (c) encourages both appropriate clinical use and new research into hormone therapies [31].

## 5. Estrogen, HRT, and Benefits to Women’s Health

It is well established that estrogen not only enhances skin health [7,9,17,34] but also has a positive influence on many age-related disorders [20,35,36,37,38]. Developing evidence suggests that HRT may confer cardioprotective effects [37] and reduce the risk of dementia [38]. HRT treatment of vasomotor symptoms reduced hot flashes by approximately 60–90% [39,40]. Notably, a systematic review and meta-analysis published in *JAMA* focusing on women aged 50–59 years found that initiation of HRT during midlife was associated with a reduced risk of dementia [41], a finding further supported by observational studies and reviews [42,43,44].

More recent reports suggest that estrogen administration utilizing the “timing hypothesis”, which states that estrogen therapy should be administered shortly after the onset of menopause (according to age and/or time since menopause), demonstrated improvements in skin health, bone density (osteoporosis), brain function (cognitive, mood, and Alzheimer’s disease risk), muscle mass, and cardiovascular health, along with metabolism to mitochondrial energy function [20,45,46,47].

**Figure 3:**
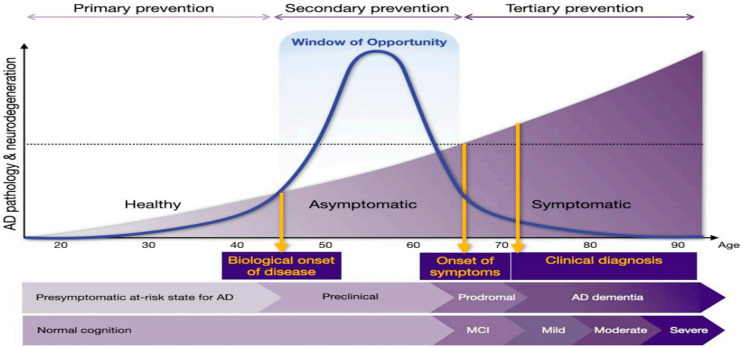
Progression of Alzheimer’s disease (AD) and the timing of preventive interventions. The diagram illustrates the progression of AD from a healthy state through to severe dementia, highlighting the stages at which primary, secondary, and tertiary preventive interventions can be applied. The curve represents the progression of AD pathology and neurodegeneration over time, beginning from an unaffected state, transitioning through the asymptomatic stage during which the disease is underway but symptoms are not yet evident, and progressing to the symptomatic and final stages. The vertical lines indicate critical junctures in disease progression: the biological onset of disease, estimated to occur in midlife; the onset of symptoms, marking the transition from asymptomatic to symptomatic phases; and the typical age of clinical diagnosis, around age 72. The primary, secondary, and tertiary prevention phases represent the spectrum of interventions. Primary prevention targets the earliest stage, aiming to prevent the onset of AD pathology; secondary prevention focuses on early detection and intervention during the preclinical AD phase to halt pathological accumulation and delay the onset of symptoms; and tertiary prevention involves managing existing disease to mitigate severity and improve quality of life in patients. HRT or menopause hormone therapy (MHT), initiated in midlife, is classified within the spectrum of primary to secondary prevention. Highlighted across the transition from healthy to early symptomatic stages, the “window of opportunity” delineates the critical period for preventive measures and disease-modifying treatments to potentially halt or slow the progression of AD. MCI = mild cognitive impairment. Adapted from Mosconi et al. 2025 [48] by license to ED Lephart 1624146-2 from RightsLink, Copyright Clearance Center.

In this regard, the “timing hypothesis” may have two perspectives. One suggests a window of HRT in recently menopausal women [31]. The other perspective is to consider HRT as a transdermal application during perimenopause, which usually occurs between 40 and 50 years of age, that would provide protection against hot flashes and night sweats and support the transition of age-related disease prevention until menopause takes place [44]. However, a review by Aarit et al. in 2022 suggested that the menopausal transition is associated with a significant decline in brain energy metabolism, increasing vulnerability to Alzheimer’s disease [47]. Consequently, a “preventive window or window of opportunity” has been identified during the fourth and fifth decades of life, when the brain undergoes substantial yet potentially reversible changes, making this period optimal for implementing risk-reduction strategies for Alzheimer’s disease in women [48] (see Figure 3). Furthermore, Mosconi et al.’s research indicates that initiating hormone replacement therapy (HRT) early—at the onset of menopausal symptoms—may provide neuroprotective benefits, with evidence showing a 32% lower risk of dementia among women who used estrogen-only therapy during midlife [48].

As a result of the transformation in scientific information on the perspective of HRT use in women, it is proposed that the treatment of women for vasomotor symptoms with HRT in clinical trials also include secondary endpoints that may include skin health, cardiovascular parameters, and brain function (especially to determine the impact on the risk of Alzheimer’s disease). This would also support Mosconi’s proposal that the “window of opportunity” begin earlier than the onset of menopause to implement risk-reduction strategies to lower Alzheimer’s disease risk, onset, and progression [48]. Of course, limitations and contraindications would include patients with a history of thromboembolism and hormone-sensitive cancer [31,49]. But the benefits of large-scale clinical trials would be a boon for women’s health to determine, in a meaningful way, a new view of HRT treatments and a broader view of hormone therapy advancements.

## 6. HRT in Perimenopausal and Menopausal Patients: A Proposal

In recent reviews, it has been proposed that HRT may be an option as an esthetic treatment for women during perimenopause and menopause [50,51,52]. While several other options are covered in these reviews, such as topical estrogen therapy, topical retinoids, topical cosmeceuticals, dermal injectables, biostimulators, and energy-based treatments (lasers and radiofrequency devices) [34,51], this represents a paradigm shift from an interdisciplinary consensus of gynecologists, plastic surgeons, and dermatologists for the use of HRT as an option for cosmetic treatment in aging women [51]. These reports update and advance the perspective of HRT use for skin health, which is not new, since many clinical studies have been conducted in this area with positive results. For example, in earlier studies by Wolff and colleagues at Yale University in 2005 reported that 9 postmenopausal women who had used HRT for five years showed fewer wrinkles and less skin rigidity compared to 11 postmenopausal women who had not used HRT, and that those women who started HRT soon after menopause showed the greatest benefits [53]. In 2007, Sator et al., in a prospective, randomized, double-blind, placebo-controlled trial, found that combined 17β-estradiol and progestogen therapy in 40 postmenopausal women (after 7 months of treatment) increased skin elasticity and thickness, along with enhancing hydration, compared to baseline measurements [54].

These earlier studies were supported by more recent reports and reviews that examined the positive influences of HRT on collagen, skin thickness, elasticity, hydration, and wound healing [34,50,51,55]. Also, in 2023, Pivazyan et al. examined skin rejuvenation in women using HRT via a systematic review and meta-analysis [56]. The Pivazyan et al. report used 15 studies comprising 1589 patients that analyzed skin elasticity, collagen content, and skin dryness. This meta-analysis reported that HRT (or MHT) increased collagen content and skin elasticity, thereby reducing the severity of wrinkles and increasing skin thickness and hydration [56]. The authors concluded from this systematic review and meta-analysis that “HRT can be used not only to treat symptoms of menopause but also to become a new direction in the rejuvenation and revitalization of the skin in women during menopause” [56].

It should be noted that HRT is not currently approved for esthetic treatment by the US FDA, and all hormonal and related therapeutic interventions require a careful evaluation of the risks and benefits through shared decision-making between the patient and healthcare professionals [31,49,51]. Several ethical considerations come into play, including (a) medical necessity vs. dermal enhancement; (b) non-maleficence since HRT affects multiple tissue/organ systems and risks [thromboembolism, breast or endometrial cancer (depending on formulation), cardiovascular, metabolic and hormonal effects]; (c) informed consent with patients understanding that HRT is not a cosmetic treatment; (d) scope of practice and competence where HRT is traditionally managed by endocrinologists/gynecologists, etc.; and (e) the potential conflicts of interest for the clear separation between medical judgment and marketable interest. Prescribing HRT for esthetic benefit is not inherently unethical, but it carries a higher ethical burden than traditional dermatological interventions [52].

Of course, the role of topical and oral therapies for estrogen-deficient skin has also been proposed [57,58,59,60]. However, natural plant-derived estrogenic compounds like isoflavonoids, when tested, were not as effective compared to 17β-estradiol [58,61].

Finally, one additional point must be made. The U.S. Food and Drug Administration traces its origins to the Pure Food and Drugs Act of 1906, which gave the federal government authority to regulate food and medicines sold across state lines (the agency itself was formally named the FDA in 1930). When these new regulations took effect, many popular patent medicines were forced off the market. Topical estrogen creams—widely sold at the time for a broad range of “female complaints” are often cited as among the best-selling products that disappeared under the new regulatory framework, illustrating how sweeping the impact of the law was on the early pharmaceutical marketplace [62].

## 7. Conclusions

As women age, especially during perimenopause and menopause, their estrogen levels decline, leading to widespread effects because estrogen influences many body/organ systems—not just reproduction [2,20]. With estrogen decline, common aging-related symptoms may include hot flashes, night sweats, sleep disturbance, mood changes, brain fog, vaginal dryness, loss of bone density, increased cardiovascular and Alzheimer’s disease risk, etc. [2,44,48,63]. HRT aims to restore estrogen (and sometimes progesterone) levels to relieve symptoms and reduce long-term health risks [2,20,44,48,63]. However, while several treatments are available to address or ameliorate the decline of skin health with aging, a paradigm shift has taken place where interdisciplinary medical consensus and recommendations for treating perimenopausal and menopause patients may include HRT [50,51,52]. Of course, this is just a proposal that has been put forth in various recent publications in review articles that need to be evaluated very carefully. All hormonal and all numerous other treatments require a review of the risks/benefits to be discussed among the patient and medical professionals to determine the most effective interventions for treating hormone-related skin changes, which may include a) age, the perimenopause interval, and time since menopause; b) type, dose, and route of hormones (oral vs. transdermal); and c) personal and family history (e.g., history of hormone-sensitive cancer and clotting risk) [1,2,31,49,51]. Finally, the use of HRT for treating hormone-related skin changes requires further investigation and validation.

## 8. Strengths and Limitations

This narrative overview provides a readable, thoughtful, and practical exploration of the critical combination of factors, such as aging, the decline and loss of estrogen in women during perimenopause and menopause, and the potential for HRT to be utilized on an individualized risk–benefit approach not only for symptomatic women but possibly for esthetic treatment that could lead to changes not only to improve skin health but where “estrogen can assist a women’s body to operate at its best”, according to the US FDA [31]. The review authors advance this new perspective by describing and interpreting the literature in the field, as well as by applying their training and experience in these research areas, which required detailed and integrative interpretations of complex focal components while maintaining a broad perspective [64,65]. This narrative overview does not aim to be a systematic synthesis that answers specific, highly focused questions; instead, it offers an expansive perspective joining together a body of research knowledge to propose novel strategies to delay the onset of and/or slow down the progression of estrogen loss in women through HRT, where appropriate. This overview does not provide an exhaustive, critical/comprehensive review of the literature; however, the usefulness of this article is that it can stimulate new ideas and discussion among physicians and scientists with various backgrounds/training, including gynecologists, neuroscientists, and dermatologists, in the future.

## 9. Future Directions

Today, HRT has evolved beyond its traditional applications. In 2016, the American College of Obstetricians and Gynecologists recommended low-dose topical estrogen administration for the treatment of vaginal dryness and atrophy [66], and its use has expanded into dermatologic applications. In 2023, at the Menopause Society meeting, the use of local estrogen cream applied to the face was presented as an effective treatment for aging skin. This development has led several cosmetic and dermatology companies to provide topical estrogen formulations as extemporaneously compounded products for esthetic use [67]. Moreover, therapeutic approaches to managing the perimenopausal and menopausal transition periods constitute important dermatologic interventions. In this context, the association between topical estrogen use for skin rejuvenation and other estrogen-dependent processes, such as dermal white adipose tissue depletion, highlights several relevant mechanistic pathways worthy of consideration [68,69,70]. For example, AI technology is rapidly transforming the management of menopausal health, particularly in dermatology, with the potential to provide highly personalized and data-driven symptom management solutions that bridge gaps in research on the physical and hormonal changes associated with menopause [71,72,73,74]. Finally, HRT can be used to improve skin parameters in menopausal women by addressing collagen loss, dryness, and reduced elasticity [51,52,55]. Nevertheless, this hormonal intervention requires further research and justification.

## Figures and Tables

**Table 1:** Benefits of 17β-estradiol in skin health.

*Validation of the following parameters as influenced by 17β-Estradiol*	Reference
1. enhances skin quality	[7,8,9,19]
2.↑ thickness, turgor, firmness, tone, barrier function	[7,8,9,19]
3. ↑ immune function	[7,8,9,20]
4. ↑ hydration	[7,8,9,19,20]
5. enhances wound healing	[7,8,9,19,20]
6. ↑ blood flow	[7,8,9]
7. ↑ elasticity	[7,8,9,18,19]
8. protects against photo-aging	[7,8,9,18,19]
9. ↑ fibroblast function	[7,8,9,19]
10. ↑ antioxidant production	[7,8,9]
11. ↑ vitamin D production (via vitamin D-binding protein)	[7,8,9]
12. ↓ wrinkles	[7,8,9,19,20]
13. ↓ matrix metalloproteinases (MMPs)	[7,8,9,19,20]
14. ↓ oxidative stress	[7,8,9,14,19]
15. ↓ androgen hormone action	[7,9]
16. maintains skin homeostasis	[7,9]
17. modulates epidermal keratinocyte proliferation	[7,9]
18. ↑ transforming growth factor beta (TGF-β)	[7,9]
19. ↑ expression of estrogen receptor beta	
***Presumably or likely parameters influenced by 17β-Estradiol***	
20. enhances sebaceous gland function	[7,9]
21. ↓ pore size	[7,9]
22. enhances skin and gastrointestinal (GI) microbiome	[21,22,23]
23. enhances facial attractiveness and perceived age	[24,25]
24. enhances psychological and self-perception	[24,25]

Adapted from Ref. [9], with permission from Springer/Nature publishers. Up arrow indicates increase; down arrow indicates decrease.

**Table 2:** Characteristics of the Women’s Health Initiative (2002–2004) report compared to today’s perspective (2020s).

Parameter	WHI Findings (2002)	Today’s Perspective (2020s)
Who was Studied	Average age 63; many women were 10 plus years post-menopause	Age and time since menopause are now critical factors
Hormone Therapy Studied	Oral conjugated equine estrogen (CEE) + medroxyprogesterone acetate (MPA)	Many better options now: transdermal estrogen, micronized progesterone, lower doses
Heart Disease	Increased coronary heart disease risk (combined therapy)	Timing hypothesis: starting HRT before age 60 or within 10 years of menopause may be protective
Stroke	Increased risk	Risk depends on dose, route, and age; transdermal estrogen shows lower risk
Blood Clots (VTE)	Increased risk	Linked to oral estrogen; transdermal routes have much lower risks
Breast Cancer	Slightly increased risk with estrogen plus progestin; decreased risk (estrogen only)	Risk varies by type of progesterone, duration, and individual risk factors
All-cause Mortality	No overall benefit	Reduced mortality when HRT is started in younger recently menopausal women
Bone Health	Reduced fractures	Still considered effective for osteoporosis prevention
Cognitive Health	Increased dementia risk, women > age 65	Increasing evidence that HRT may be protective in younger recently menopausal women
Menopausal Symptom Relief	Not a primary focus	HRT effective treatment for hot flashes/night sweats
Overall Conclusion	HRT is harmful, public perception-dangerous	Individualized risk–benefit approach beginning to be standard of care

Takeaways (plain language): (a) WHI results were overgeneralized to all women; (b) age, timing, dose, and formulation(s) matter in risk/benefit evaluation; (c) HRT is now considered safe and beneficial for many women, especially those under 60 years of age, within 10 years of menopause, and using modern hormone formulations that had good benefits and lower risks. VTE = venous thromboembolism. Data and information in this Table were generated from the following references [1,28,29,30,31,32,33].

## Data Availability

The data cited in this overview are contained within the publications referenced.

## Abbreviations

The following abbreviations are used in this manuscript:

β	beta
AD	Alzheimer’s disease
GI	gastrointestinal
HRT	hormone replacement therapy
JAMA	Journal of the American Medical Association
MHT	menopause hormone therapy
MMP	matrix metalloproteinase
ROS	reactive oxygen species
US FDA	United States Food and Drug Administration
TGF-β	transforming growth factor beta
WHI	Women’s Health Initiative
